# The relationship between weight-adjusted-waist index and suicidal ideation: evidence from NHANES

**DOI:** 10.1007/s40519-024-01666-4

**Published:** 2024-05-14

**Authors:** Shijie Guo, Guangwei Qing, Qiqi Chen, Guang Yang

**Affiliations:** 1 Department of Applied Social Sciences, The Hong Kong Polytechnic University, Kowloon, Hong Kong SAR China; 2 Jiangxi Mental Hospital & Affiliated Mental Hospital of Nanchang University, Third Clinical Medical College of Nanchang University, Nanchang, Jiangxi China; 3 Department of Neurology, Kunshan Hospital of Traditional Chinese Medicine, Suzhou, Zhejiang China

**Keywords:** Waist-to-weight index, Suicidal ideation, Mental health assessments, Cross-sectional analysis, Predictive accuracy

## Abstract

**Background:**

Amidst growing evidence of the intricate link between physical and mental health, this study aims to dissect the relationship between the waist-to-weight index (WWI) and suicidal ideation within a representative sample of the US population, proposing WWI as a novel metric for suicide risk assessment.

**Methods:**

The study engaged a sample of 9500 participants in a cross-sectional design. It employed multivariate logistic and linear regression analyses to probe the association between WWI and suicidal ideation. It further examined potential nonlinear dynamics using a weighted generalized additive model alongside stratified analyses to test the relationship's consistency across diverse demographic and health variables.

**Results:**

Our analysis revealed a significant positive correlation between increased WWI and heightened suicidal ideation, characterized by a nonlinear relationship that persisted in the adjusted model. Subgroup analysis sustained the association's uniformity across varied population segments.

**Conclusions:**

The study elucidates WWI's effectiveness as a predictive tool for suicidal ideation, underscoring its relevance in mental health evaluations. By highlighting the predictive value of WWI, our findings advocate for the integration of body composition considerations into mental health risk assessments, thereby broadening the scope of suicide prevention strategies.

## Introduction

There is a genuinely severe philosophical problem, and that is suicide [1], a profound societal crisis. Annually, approximately 700,000 individuals end their own lives [2]. According to the US Centers for Disease Control and Prevention (CDC), in 2021, there were 48,183 suicides in the US, a figure that escalated to 49,449 in 2022, indicating a 2.6% increase [3]. The complexity of suicide prevention is attributed to its multifaceted causes, including societal (e.g., familial responsibilities), political (e.g., policies on minimum wage), cultural (e.g., stereotypes of masculinity), and economic factors (e.g., unemployment, lower socioeconomic status) [4]. Identified risk factors encompass mental health disorders, substance abuse, chronic pain, a history of suicide in the individual or family, experiences of aggression, access to firearms at home, and recent discharge from incarceration [5–9]. Additionally, witnessing suicidal behaviors in others can heighten suicide risk [10]. Despite demographic variances among groups such as adolescents, sexual minorities, and adults, research consistently shows that individuals harboring suicidal thoughts face a greater risk of suicide completion than those without such thoughts [11–14]. Despite efforts across medical, socioeconomic, and political domains to mitigate and prevent suicide, a significant reduction in the prevalence of suicidal ideation remains elusive [15], highlighting the critical need for an in-depth examination of suicidal ideation.

Obesity represents a widespread, severe, and financially burdensome chronic condition affecting both adults and children. In 2022, the CDC reported that the prevalence of adult obesity in the US had escalated, with 22 states having an adult obesity prevalence at or above 35%, an increase from 19 states in 2021[16]. Obesity is a significant risk factor for a variety of severe health conditions, including hypertension, cardiovascular diseases, diabetes mellitus, asthma, joint disorders, cholelithiasis, as well as gallbladder disease. [5, 17, 18]. Doctors believe that obesity is a complex, heterogeneous chronic disease that manifests differently in patients and, therefore, requires individualized long-term management [19]. Consequently, identifying accurate and reliable metrics for evaluating obesity is critically essential.

Body mass index (BMI) has long been a widely accepted standard for identifying levels of obesity. However, BMI cannot distinguish excess fat, muscle, or bone mass. BMI cannot differentiate between locations of fat [20, 21]. It is generally accepted that abdominal (visceral) fat is more closely associated with health risks than fat in other body parts [22–25]. The weight-adjusted-waist index (WWI) represents a novel metric for obesity assessment, demonstrating enhanced precision in quantifying lean muscle and adipose tissue mass when juxtaposed with traditional measures such as BMI and waist circumference (WC) [26, 27]. The literature suggests that WWI is associated with a variety of conditions, including fatty liver, cognitive function, hyperuricemia, diabetes mellitus, kidney stones, dyslipidemia, and urinary incontinence [28, 27, 29–33]. It may serve as a more accurate predictor of risk.

Several studies identified associations between obesity and suicide risk, depressive symptoms, and mood problems [34–37], and they have all used BMI as an indicator for determining obesity. Notwithstanding, the relationship between WWI and the prevalence of suicidal ideation has not yet been investigated. Consequently, the present study seeks to elucidate the potential correlation between WWI and suicidal ideation within the representative sample of the US population, employing data sourced from the National Health and Nutrition Examination Survey (NHANES) from 2013 to 2018.

## Methods

### Data source and study participants

Our investigation leveraged datasets obtained from NHANES, a project spearheaded by the National Center for Health Statistics (NCHS) in the United States. This comprehensive survey collates physical assessments and interview responses to gather extensive data on demographics, socioeconomic status, and health-related information. Focused on the NHANES data from 2013 to 2018, our study sought to secure a current and emblematic sample of the US population. The data acquisition process was governed by stringent ethical protocols, including the endorsement from the NCHS's Research Ethics Review Board and the acquisition of informed, written consent from all contributors. This groundwork enables our examination of health patterns and supports formulating informed health policy recommendations.

**Fig. 1:**
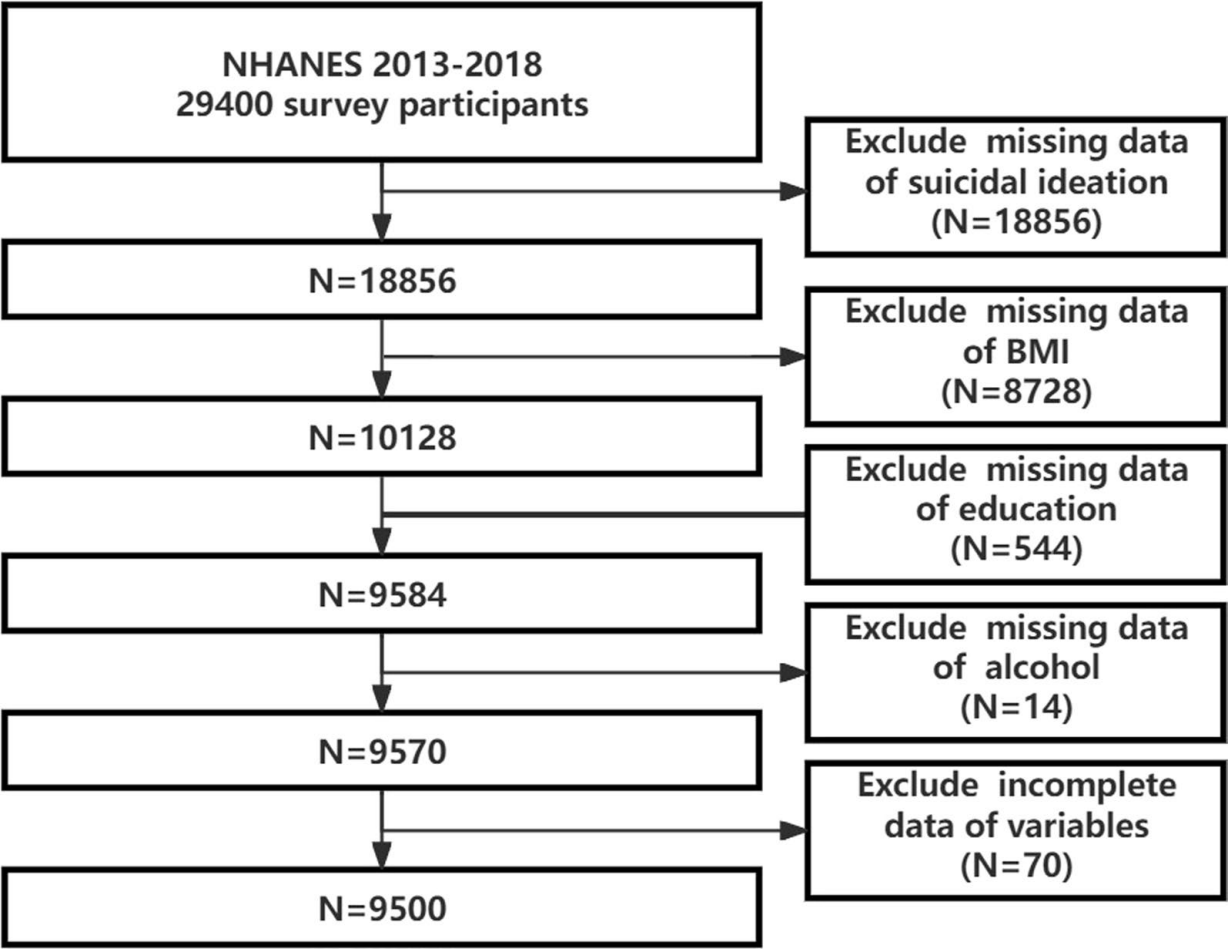
Flowchart of participants selection. NHANES, National Health and Nutrition Examination Survey

In this investigation, we employed a stratified, multistage probability sampling approach to ensure the comprehensive inclusion of participants. The initial participant pool comprised 29,400 individuals enrolled in the NHANES from 2013 to 2018. After excluding participants with incomplete data sets (*N* = 19,900), the analytical sample was refined to 9500 individuals (Fig. 1).

### Definition of exposure variable

WWI is an anthropometric measure to assess central obesity. It derives from an individual’s WC and body weight. Each participant’s WWI is calculated using a specific formula: WC divided by the square root of body weight, with the outcome rounded to two decimal places (WWI = WC/√body weight, where WC is measured in cm and body weight in kg).

### Assessment of suicidal ideation

Within the context of the survey’s Mental Health—Depression Screener segment, participants responded to a critical inquiry aimed at evaluating the presence of suicidal ideation: “Over the last two weeks, how often have you been troubled by thoughts of being better off dead, or by thoughts of self-harm?” Responses indicating such thoughts on “Several days”, “More than half the days”, or “Nearly every day” were interpreted as markers of suicidal ideation.

### Covariates

We incorporated a comprehensive set of covariates: age, sex, race, education, marital status, smoking status, alcohol consumption, hypertension, diabetes, angina pectoris, arthritis, cancer, and asthma. Education was divided into two categories: individuals with qualifications higher than high school. Smoking status encompassed those who have smoked at least 100 cigarettes in their lifetime. Alcohol consumption was defined as having at least 12 alcoholic beverages annually.

### Statistical analysis

We meticulously analyzed the demographic characteristics of participants, differentiated by the presence of suicidal ideation, employing weighted Chi-square tests for categorical variables and *t*-tests for continuous ones. The relationship between WWI and suicidal ideation was explored through multivariate logistic regression analyses. In contrast, the correlation between WWI and the level of suicidal ideation was examined using multivariate linear regression analyses. To uncover any potential nonlinear dynamics between WWI and suicidal ideation, we applied a weighted generalized additive model complemented by smooth curve fitting techniques. Additionally, stratified analyses and interaction tests were conducted to investigate the consistency of the WWI–suicidal ideation relationship across various subgroups. On a two-tailed basis, a *p*-value of less than 0.05 indicated statistical significance. All statistical analyses were performed using R Statistical Software (Version 4.2.2, available at http://www.R-project.org, developed by The R Foundation) and the Free Statistics analysis platform (Version 1.8, based in Beijing, China).

## Results

### Characteristics of the study participants

A total of 9500 participants were included in our research, with 9173 not exhibiting suicidal ideation and 327 reporting suicidal ideation. The overall prevalence of suicidal ideation was 3.44% (weighted proportion) (Table 1). A key finding from Table 1 is the statistically significant difference in WWI between groups (*p* < 0.001), with individuals exhibiting SI having a higher mean WWI than those without. This suggests that WWI may be a relevant factor in understanding suicidal ideation. Conversely, BMI does not significantly differ between groups (*p* = 0.401).

**Table 1:** Basic characteristics of participants

Variables	Without SI^a^ (*n* = 9173)	With SI^a^ (*n* = 327)	*p*
Age, years	47.55 ± 16.94	47.72 ± 17.29	0.903
Sex			0.100
Male	49.06	54.79	
Female	50.94	45.21	
Race/ethnicity, *n* (%)			0.405
Mexican American	15.02	17.37	
Other Hispanic	11.10	8.29	
Non-Hispanic White	39.32	43.28	
Non-Hispanic Black	20.17	19.36	
Other races	14.39	11.71	
Education level, *n* (%)			0.802
≤ High school	43.44	44.10	
> High school	56.56	55.90	
Marital status, *n* (%)			0.267
Married	51.90	54.05	
Widowed	6.50	7.75	
Divorced	10.91	13.72	
Separated	3.43	2.40	
Never married	18.78	12.49	
Living with partner	8.48	9.57	
Smoking, *n* (%)			0.945
No	54.71	54.38	
Yes	45.29	45.62	
Alcohol, *n* (%)			0.683
No	29.02	27.64	
Yes	70.98	72.36	
Hypertension, *n* (%)			0.506
No	64.41	62.63	
Yes	33.24	34.59	
Diabetes, *n* (%)			0.3215
No	86.95	84.86	
Yes	13.05	15.14	
Angina pectoris, *n* (%)			0.532
No	97.53	96.74	
Yes	2.47	3.26	
Arthritis, *n* (%)			0.917
No	74.03	73.66	
Yes	25.97	26.34	
Cancer, *n* (%)			0.501
No	90.34	88.72	
Yes	9.66	11.28	
Asthma, *n* (%)			0.053
No	84.60	90.88	
Yes	15.40	9.12	
WWI	11.02 ± 0.83	11.31 ± 0.86	< 0.001
BMI (kg/m^2^)	29.22 ± 6.92	29.60 ± 6.99	0.401

^a^*SI* suicidal ideation, *WWI* weight-adjusted-waist index

### Association between WWI and suicidal ideation

**Fig. 2:**
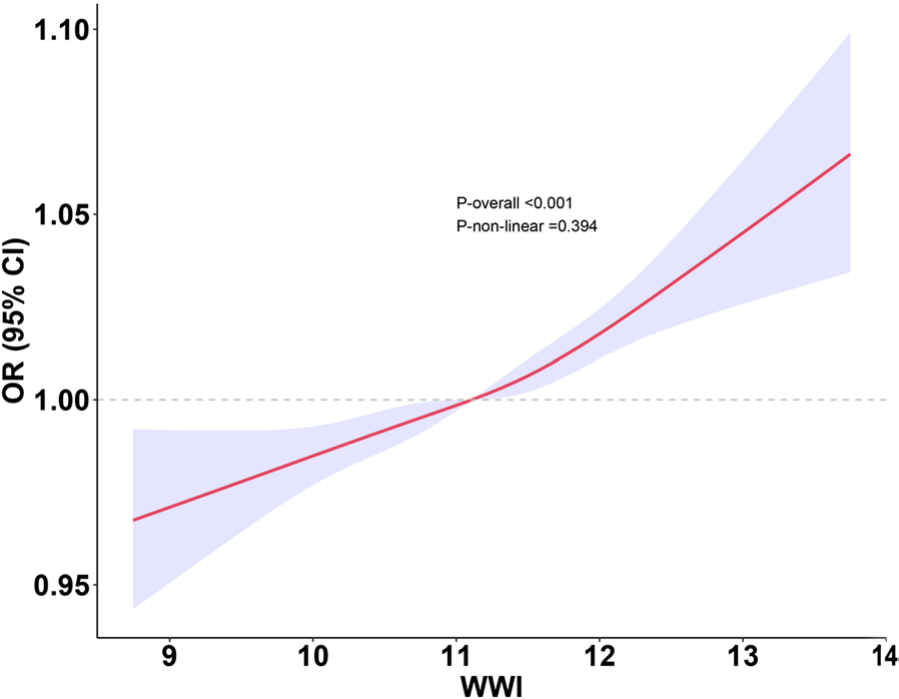
The association between WWI and suicidal ideation. The solid red line represents the smooth curve fit between variables. The shadow area represents the 95% confidence interval from the fit: weight-adjusted-waist index, WWI

**Fig. 3:**
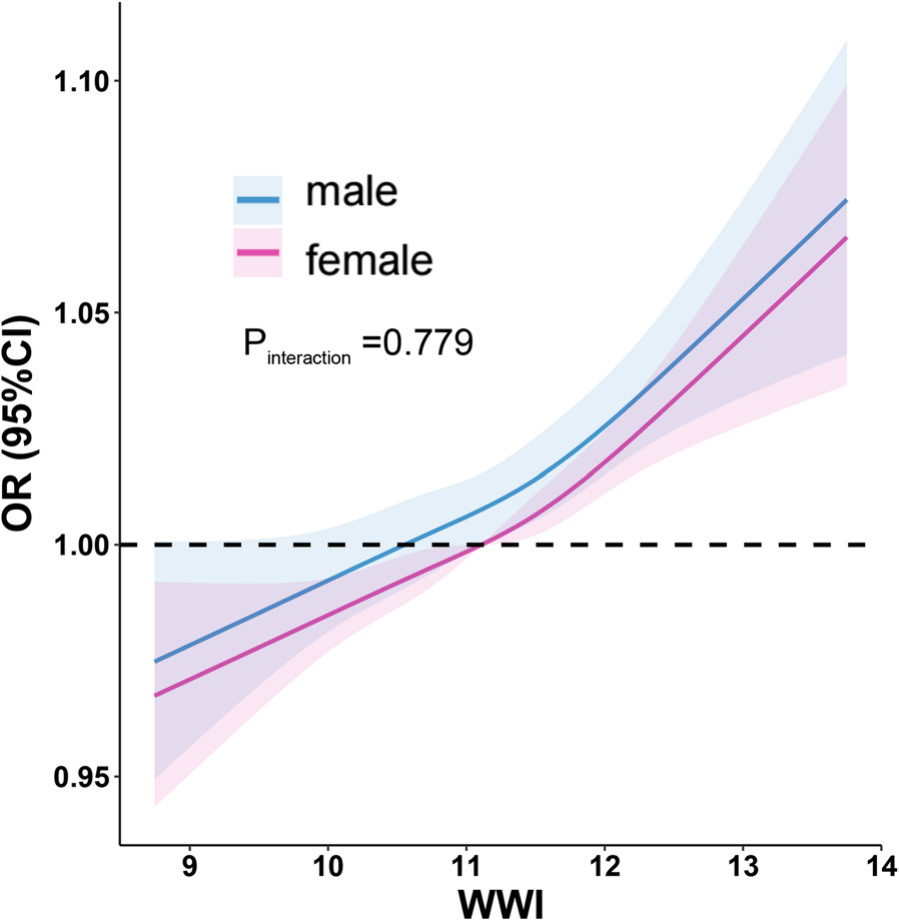
The association between WWI and suicidal ideation of different genders. The solid red line represents the smooth curve fit of females between variables. The solid blue line represents the smooth curve fit of males between variables. The shadow area represents the 95% confidence interval from the fit: weight-adjusted-waist index, WWI

Table 2 details the results from multivariate logistic regression analyses conducted using two distinct models. These analyses identified a strong and statistically significant positive association between WWI and the likelihood of suicidal ideation, consistent across both models. After comprehensive adjustment, individuals with a one-unit increase in WWI were found to have a 67.9% heightened risk of experiencing suicidal ideation [OR: 1.679, 95% CI 1.532–1.969]. Moreover, when analyzing WWI by quartiles, those in the highest quartile of WWI faced a risk increase of 1.761 times compared to individuals in the lowest quartile [OR: 2.761, 95% CI 2.062–3.698], as shown in Table 2. A generalized model incorporating smooth curve fitting was also utilized to validate the nonlinear association between WWI and suicidal ideation. The analysis confirmed a nonlinear positive relationship between WWI and suicidal ideation (Fig. 2), with the observed patterns remaining consistent across both genders (Fig. 3).

**Table 2:** Association between WWI and suicidal ideation

Characteristics	Model 1 [OR (95% CI)]	Model 2 [OR (95% CI)]
Continuous WWI	1.521 (1.324, 1.747)	1.679 (1.532, 1.969)
*p-value*	< 0.001	< 0.001
Quartile		
Q1	Reference	Reference
Q2	1.653 (1.154, 2.370)	1.653 (1.154, 2.370)
Q3	1.798 (1.122, 2.884)	1.798 (1.122, 2.884)
Q4	2.761 (2.062, 3.698)	2.761 (2.062,3.698)
*p* for trend	< 0.001	< 0.001

Model 1: not adjusted for covariates

Model 2: adjusted for age, sex, race, education level, marital status, smoking, alcohol, hypertension, diabetes, angina pectoris, arthritis, cancer, and asthma

### Association between WWI and the level of suicidal ideation

Table 3 elucidates the significant association between WWI and the level of suicidal ideation, treating both variables as continuous. Initial findings from the unadjusted Model 1 reveal an exponentiated coefficient of 2.770 (95% CI 1.011–1.028), highlighting a statistically significant positive relationship. The adjusted Model 2, which considers a range of health behaviors and conditions, presents an exponentiated coefficient of 1.023 (95% CI 1.012–1.035), indicating a significant link between higher WWI and increased suicidal ideation levels (*p* < 0.01). Quartile analysis further corroborates these findings, with the highest WWI quartile (Q4) demonstrating a notably elevated risk (*p* for trend < 0.001). These insights underscore the vital connection between WWI and the severity of suicidal thoughts.

**Table 3:** Association between WWI and the level of suicidal ideation

Characteristics	Model 1 [Exponentiated coefficients (95% CI)]	Model 2 [Exponentiated coefficients (95% CI)]
Continuous WWI	2.770 (1.011, 1.028)	1.023 (1.012, 1.035)
*p*	< 0.001	< 0.01
Quartile		
Q1	Reference	Reference
Q2	1.017 (1.003, 1.033)	1.021 (1.002, 1.040)
Q3	1.025 (1.002, 1.049)	1.031 (0.002, 1.061)
Q4	1.048 (1.028, 1.068)	1.057 (1.031, 1.082)
*p* for trend	< 0.001	< 0.001

Model 1: not adjusted for covariates

Model 2: adjusted for age, sex, race, education level, marital status, smoking, alcohol, hypertension, diabetes, angina pectoris, arthritis, cancer, and asthma

### Subgroup analyses

To assess the uniformity of the association between WWI and suicidal ideation across the representative sample of the US population and to pinpoint any parameters specific to subpopulations, we undertook a detailed subgroup analysis alongside interaction tests. Table 4 examines the odds ratio (OR) for suicidal thoughts across different demographics and health conditions, indicating no significant interaction effects based on sex, age, education level, smoking status, alcohol consumption, hypertension, diabetes, angina pectoris, arthritis, cancer, or asthma. The odds ratios suggest a relatively consistent association across these subgroups, with variations in the magnitude of risk but no statistically significant differences in the pattern of association. In parallel, Table 5 explores the exponentiated coefficient for the level of suicidal ideation across similar subgroups, again showing no significant interaction effects, which implies a uniform relationship between WWI and the severity of suicidal ideation across different demographic and health-related categories.

**Table 4:** Subgroup analysis of the relationship between WWI and suicidal ideation

Subgroup	OR (95% CI)	*p* for interaction
Sex		0.585
Male	1.53 (1.27, 1.85)	
Female	1.54 (1.25, 1.88)	
Age, years		0.497
20–44	1.35 (1.08, 1.68)	
45–59	2.12 (1.57, 2.84)	
≥ 60	1.44 (1.15, 1.81)	
Education level		0.944
≤ High school	1.45 (1.18, 1.78)	
> High school	1.61 (1.33, 1.94)	
Smoking		0.277
No	1.71 (1.42, 2.06)	
Yes	1.35 (1.08, 1.68)	
Alcohol		0.248
No	1.56 (1.19, 2.04)	
Yes	1.53 (1.30, 1.80)	
Hypertension		0.502
No	1.61 (1.35, 1.92)	
Yes	1.41 (1.13, 1.77)	
Diabetes		0.118
No	1.50 (1.28, 1.76)	
Yes	1.71 (1.25, 2.33)	
Angina pectoris		0.346
No	1.56 (1.35, 1.80)	
Yes	0.73 (0.25, 2.15)	
Arthritis		0.688
No	1.58 (1.33, 1.87)	
Yes	1.43 (1.13, 1.83)	
Cancer		0.624
No	1.58 (1.36, 1.83)	
Yes	1.20 (0.75, 1.92)	
Asthma		0.711
No	1.51 (1.30, 1.76)	
Yes	1.80 (1.19, 2.73)

**Table 5:** Subgroup analysis of the relationship between WWI and the level of suicidal ideation

Subgroup	Exponentiated coefficients (95% CI)	*p* for interaction
Sex		0.271
Male	1.030 (1.010, 1.041)	
Female	1.020 (1.010, 1.030)	
Age, years		0.685
20–44	1.010 (1.000, 1.030)	
45–59	1.041 (1.020, 1.062)	
≥ 60	1.020 (1.010, 1.030)	
Education level		0.928
≤ High school	1.020 (1.010, 1.030)	
> High school	1.020 (1.010, 1.030)	
Smoking		0.591
No	1.030 (1.020, 1.041)	
Yes	1.010 (1.000, 1.030)	
Alcohol		0.673
No	1.020 (1.010, 1.030)	
Yes	1.020 (1.010, 1.030)	
Hypertension		0.24
No	1.030 (1.020, 1.030)	
Yes	1.010 (1.000, 1.030)	
Diabetes		0.094
No	1.020 (1.010, 1.030)	
Yes	1.030 (1.010, 1.051)	
Angina pectoris		0.264
No	1.020 (1.010, 1.030)	
Yes	0.970 (0.905, 1.030)	
Arthritis		0.863
No	1.020 (1.010, 1.030)	
Yes	1.020 (1.000, 1.030)	
Cancer		0.614
No	1.020 (1.010, 1.030)	
Yes	1.010 (0.990, 1.030)	
Asthma		0.625
No	1.020 (1.010, 1.030)	
Yes	1.020 (1.000, 1.030)	

## Discussion

We sought to elucidate the association between WWI and the propensity for suicidal ideation within the representative sample of the US population. Conducting a comprehensive cross-sectional analysis with 9500 participants, we discerned a significant correlation that elevated WWI was significantly associated with increased suicidal ideation and increased levels of suicidal ideation. This study unveiled a nonlinear positive correlation between WWI and the incidence of suicidal thoughts, a relationship that persisted even after complete adjustments in our model. Subsequent subgroup analyses revealed that the predictive validity of WWI for suicidal ideation remained unaffected across various demographic and health-related factors, including smoking status, alcohol intake, and the presence of chronic conditions such as hypertension, diabetes, and asthma. These insights collectively affirm the potential of WWI as an efficacious predictor for suicidal ideation, underscoring its utility in mental health assessments.

Several studies documented that the prevalence of suicidal ideation or depressive symptoms increases with BMI [34, 35, 38, 39]. However, due to BMI's limitations, including its inability to differentiate between excess fat, muscle, or bone mass, and its failure to discern body fat distribution across different locations, some studies are beginning to experiment with other indicators. A study showed a significant relationship between WC, waist–height ratio, and suicidal ideation among US adult women. A Korean survey showed that BMI, WC, waist-to-hip ratio, and percent body fat were not significantly associated with suicidal ideation. In contrast, sarcopenia was strongly associated with an increased risk of suicidal ideation in older men [40]. It has also been shown that sarcopenic obesity is significantly associated with suicidal ideation [40]. Low height and perceived obesity are associated with suicidal ideation in Korean adolescents [41]. These studies also imply WWI's potential as a novel index of obesity. However, the validity of these indicators needs to be confirmed by more extensive clinical studies. In recent years, it has been found that WWI is a superior predictor of non-alcoholic fatty liver disease [33], low cognitive performance [27], hyperuricemia [28], diabetic kidney disease [42], kidney stones [30], stress urinary incontinence [31], and depressive symptoms [43]. WWI has excellent potential as an anthropometric indicator because of its easy computation and strong ability to forecast disease onset [44].

There may be multiple mechanisms for the interaction between obesity and suicidal ideation. First, the coexistence of obesity with other medical diseases may exacerbate the situation, or chronic health challenges may lead to increased mental stress, which in turn produces higher levels of suicidal ideation. It has been established that the coexistence of overweight or obesity and depression exacerbates the inflammatory response, leading to a worse prognosis and increased risk of suicide in adolescents with major depressive disorder [37]. In the setting of fibromyalgia and concomitant obesity, prior research points to a distinct (i.e., irrespective of depressive symptomatology and sleep quality) relationship between pain catastrophizing and suicidal thoughts [45]. Another research found that BMI is a risk factor for the development of severe anxiety symptoms in patients with severe anxiety disorder with comorbid dyslipidemia [46]. In addition, in patients with bipolar disorder, elevated BMI is associated with worsening clinical features, including higher rates of suicide, comorbidities, and core depressive symptoms. Second, obesity is associated with biochemical changes in the body, which may impact mood and behavior. It has been shown that obesity is associated with elevated levels of inflammation in the body [47–49], and inflammation is thought to be a possible mechanism that leads to depression and other mood disorders [50]. Some researchers believe that obesity may lead to insulin imbalance, and insulin resistance has been linked to depression [51]. There is also evidence that adipose tissue produces and releases various chemicals called adipokines, which directly affect other body parts, including the brain [52]. As a result, evaluating obesity is essential to estimating the likelihood of suicidal thoughts, and this study’s results suggest that WWI may be an effective indicator of obesity.

WWI combines body weight and waist measurements for a nuanced overview of fat distribution, offering advantages over BMI and WC, which overlook crucial aspects of body composition and total weight. From a pathophysiological perspective, WWI might provide a more explicit link to metabolic and cardiovascular risks associated with obesity. Abdominal fat, as measured by WC, is a known risk factor for metabolic syndrome, type 2 diabetes, and cardiovascular disease, which may be due to adipose gene expression of the effector genes [53], its proximity to vital organs and its role in producing inflammatory adipokines [54, 55]. However, incorporating the weight component could enhance the predictive power by indicating the overall load on the body, including the strain on bones, joints, and organs [56, 57]. Furthermore, the integrated approach of WWI better reflects the adiposity-related biochemical stress on the body. This includes the effects of fat mass on cytokine production [58], insulin resistance [59], and lipid metabolism [60], all of which are crucial for understanding the physiological basis for depression and suicidal ideation linked to obesity.

Social workers can effectively utilize WWI in their practice to identify individuals more accurately at high risk of suicide. By providing psychological support and counseling, establishing support systems, promoting education on healthy lifestyles, assisting in accessing medical and nutritional resources, and providing crisis intervention and referrals, social work can not only help the obese population improve their mental health but also improve their quality of life, thus playing a pivotal role in suicide prevention.

Despite its contributions, our study is not without limitations. First, the cross-sectional nature of our analysis precludes us from elucidating the causal mechanisms and effects underlying the observed associations. Second, the potential for inaccuracies in the covariates included may impact the results' precision. Third, it is essential to note that the results of this study may not necessarily translate into clinical implications, mainly due to the large sample size, which can amplify minor variations. Fourth, our analysis does not account for the potential influence of weight reduction treatments [61] on WWI due to data constraints, which could affect the associations observed. Nonetheless, our research is strengthened by its foundation on a substantial and representative dataset derived from the NHANES, providing a robust basis for our findings. Moreover, we are the first to investigate the relationship between WWI and suicidal ideation, including a variety of subgroup analyses.

## Conclusion

Elevated WWI was significantly associated with increased suicidal ideation. Relative to BMI, WWI demonstrates enhanced predictive efficacy for suicidal ideation. This facilitates the timely identification of suicide risk by social workers. Nonetheless, additional studies are necessary to validate these preliminary outcomes.

## Data Availability

The datasets analyzed during the current study are available in the NHANES [https://www.cdc.gov/nchs/nhanes/index.htm].
